# Evaluation of The Protective Effect of Hydro-Alcoholic Extract of Raspberry Fruit on Aquaporin1 Expression in Rats Kidney Treated by Methotrexate

**DOI:** 10.22074/cellj.2016.3957

**Published:** 2017-02-22

**Authors:** Saeideh Khoshnoud, Homa Mohseni Kouchesfahani, Mohammad Nabiuni

**Affiliations:** 1 Department of Animal Biology, Faculty of Biological Sciences, Kharazmi University, Tehran, Iran; 2 Department of Cell and Molecular Biology, Faculty of Biological Sciences, Kharazmi University, Tehran, Iran

**Keywords:** Methotrexate, Nephrotoxicity, Rat

## Abstract

**Objective:**

Methotrexate (MTX) is an antimetabolite drug commonly prescribed for the various cancers and autoimmune diseases. Despite its considerable therapeutic effects, nephrotoxicity is the most important side-effect of treatment with MTX. Aquaporin1 (AQP1) is a water channel proteins which is present in mammalian kidney. Raspberry fruit with antioxidant properties is able to protect biological systems from the harmful effects of free radicals. The purpose of this study was to investigate the effect of raspberry extract on expression of AQP1 and the MTX-induced nephrotoxicity in rats.

**Materials and Methods:**

In this experimental study, 60 adult male Wistar rats were divided into nine groups including control, sham, MTX treated group [single dose of 20 mg/kg of body weight (BW) MTX at the third day], raspberry treated groups [intraperitoneal (I.P) injection of 100, 200, 400 mg/kg of BW raspberry extract for ten consecutive days], MTX and raspberry treated groups. At day 11, rats were sacrificed via chloroform inhalation and kidney tissues were fixed in formalin solution for histological and immunohistochemistry analysis. The serological assays for urea, creatinine, uric acid and interleukin-6 (IL-6) levels were also performed.

**Results:**

MTX elevated serum level of the urea, creatinine, uric acid, IL-6, renal tissue damage and decreased the AQP1 expression level. Raspberry fruit extract improved the kidney function and reduced side effects of MTX in treated rats. Expression of AQP1, in a dose dependent manner was also ameliorated, as compared to control group.

**Conclusion:**

According to the findings of this study, it can be concluded that biological activity of compounds presented in raspberry fruit extract especially anthocyanins may have chemo-protective effect on kidney function and AQP1 expression in rats treated by MTX.

## Introduction

Methotrexate (MTX), a folic acid antagonist, is widely used as a cytotoxic chemotherapeutic agent for treatment of acute lymphoblastic leukaemia, lymphoma, osteosarcoma, breast cancer, head and neck cancers and also in the treatment of non- oncologic disorders such as rheumatoid diseases and psoriasis (1,3). MTX is an antimetabolite drug, bearing an ability to block the metabolism of cells. However, despite the therapeutic effects, toxicity of MTX including nephrotoxicity as well as gastrointestinal, central nervous system, hepatic, and bone marrow toxicity may restrict its applications (4,5). 

Aquaporins (AQPs) are channel-forming transmembrane glycoproteins originally discovered due to their abilities to mediate water entry or release through cell membranes driven by a transmembrane osmotic gradient (6). The first AQP was identified in 1988 by Peter Agre and his colleagues (7). 

AQP1 is a multi-subunit oligomer that is organized as a tetrameric assembly of four identical polypeptide subunits with a large glycan attached to only one subunit (8). AQPs played indispensable physiological roles in bacteria and plants, as well as in mammalian organs such as red blood cells, kidney, eye, brain and lung, where rapid transport of water takes place (9). Up to now, 13 members of the AQP family have been recognized in mammals. Some of these members are entirely permeable to water, whereas some others transport water, urea, glycerol and perhaps other small solutes (7). Kidney is a mammalian organ with the most active fluid-transporting epithelia. Seven AQP members are expressed in different segments of the nephron (10,12). AQP1 is expressed in the apical and basolateral plasma membranes in the proximal tubule, where the majority of glomerular-infiltrated fluids are absorbed. AQP1 is also expressed in the plasma membranes of the thin descending limb of Henle (TDLH) and in the microvascular endothelium of the outer medullary descending vasa recta (OMDVR), which are the structural basis of the countercurrent multiplication mechanism of urinary concentration (11,13). 

Raspberry, belonging to the Rosaceae family with 250 species, is a commercial fruit crop widely grown in all temperate regions of the world (14,15). Black raspberries consist of compounds proved to be protective against some cancers including esophageal and colon cancer in experimental animals. Compounds presented in black raspberry include folic acid, calcium, selenium, β-sitosterol, ellagic acid, ferulic acid, quercitin and vitamins A, C and E, besides the anthocyanins; cyanidin-3-O-glucoside, cyanidin- 3-O-rutinoside, cyanidin-3- O-xylosylrutinoside and cyanidin-3-O-sambubioside (16,17). 

The aim of present study was to assess the protective effects of raspberry fruit extract on AQP1 expression in rats kidney treated by MTX. 

## Materials and Methods

### Animals

In this experimental study 60 adult male Wistar rats, weighing 170-200 g, were used throughout the study. Animals were put separately in metabolic cages in an air conditioned unit and were allowed free access to standard laboratory chow and water. A 12 hours light/dark cycle (Lights on from 08:00 to 20:00), at 22 ± 2˚C conditions was maintained. To get adapted to the environment, rats under study were housed for at least 10 days in the above-stated conditions before drug administration. 

### Methods

60 adult male Wistar rats were divided into nine groups (n=6 for each group) including control (intact), sham (normal saline 1.5 mg/ kg of body weight [BW]), MTX treated group (a single dose of 20 mg/kg of BW MTX at the third day, n=12, divided into two groups, one examined on day 6 and the other on day 11), raspberry groups (100, 200, 400 mg/kg of BW raspberry extract for ten consecutive days), MTX and raspberry groups (100, 200, 400 mg/ kg of BW raspberry extract for ten consecutive days and a single dose of 20 mg/kg of BW MTX at the third day, one hour after injection of raspberry extract). All injections were performed intraperitoneally (I.P). At day 11, rats were sacrificed by chloroform inhalation to investigate the immunohistochemistry, histological and serological changes [urea, creatinine, uric acid and interleukin-6 (IL-6) levels]. For that, blood serums were collected and kidney tissues were fixed in formalin solution. 

### Preparation of raspberry extract

Raspberry was collected from Mazandaran (Iran), dried in the shade and cleaned before extraction. For extraction, 500 g of fruit powder was weighted and dissolved in a liter of 80% ethanol. After 48 hours, it was filtered through Whatmann paper and transferred to a flask. Solvent was removed using rotary evaporator (at 70˚C with an average round). The resultant thick liquid was placed in the oven (45˚C) for two days to vapor all alcoholic solvent. Thus, from 500 g raspberry fruit, 50 g dry extract was obtained. Raspberry extracts were stored at 4˚C until use. 

### Chemicals

Primary antibody AQP1 was obtained from Abcam Company (UK). Anti-rabbit HRP/DAB Detection kit was obtained from Bethyle Company (USA). IL6 ELISA Kit (rat IL-6 platinum ELISA) was obtained from Bender Medsystems Company (Austria). Phosphate buffer saline was obtained from Gibco-Invitrogen Company (UK). Blood serum bovine, hematoxylin, poly-l-lysin and eosin were obtained from Sigma Company (USA). Xylene, Tween, Triton, formalin, paraffin, ethanol, methanol and chloroform were obtained from Merck Company (Germany). MTX was obtained from Kocak Farma (Turkey). 

### Immunohistochemistry and histological exami- nation

Rats were sacrificed via chloroform inhalation and removed kidneys were fixed in 10% formalin, embedded in paraffin and 5 μm sections were prepared and mounted on poly-l-lysin-coated glass slides (for immunohistochemistry) and gelatin- coated glass slides (for staining by hematoxylin and eosin). The expression of AQP1 was determined by immunohistochemistry. Briefly, slides were put in a 60˚C oven (Fan Azma Gostar, Iran) for 60 minutes, deparaffinized by xylene, and passed through a down series of ethanol solutions to water. All slides were treated for 15 minutes with a 1.5% H_2_O_2_solution in water to block endogenous peroxidase. Then, the sections were washed twice (3 minutes each) in phosphate buffer saline (PBS) supplemented with 0.01% Tween (PBST) and 0.02 % Triton X-100. For antigen retrieval, the slides were placed in citrate buffer, microwaved (Daewoo, England) and washed thrice (3 minutes each) in the previously presented PBS-Tween- Triton X-100 buffer. To eliminate nonspecific binding, sections were subsequently incubated with blocking solution [PBST supplemented with 1% blood serum bovine (BSA)] for 10 minutes at room temperature. The sections were then incubated (Iran khod saz, Iran) overnight at 4˚C with AQP1 primary antibody, diluted in PBST and supplemented with 0.2% BSA (diluted 1:500). The sections were then washed with free Triton buffer thrice (5 minutes each) and incubated for 60 minutes each in horseradish peroxidase conjugated goat anti-rabbit secondary antibodies (Bethyle,USA) diluted in PBST supplemented with 0.2% BSA (dilution 1:10) at 37˚C. Next, they were washed thrice (5 minutes each) in free Triton buffer, followed by 10 minutes incubation of substrate solution diaminobenzidine (DAB) at room temperature, and washing thrice (5 minutes each) in free Triton buffer and one minute incubation in substrate solution with hematoxylin. Once again, they were washed thrice (2 minutes each) in tap water. The sections rehydrated through xylenes, graded ethanol solutions to water and were examined under a light microscope (Zeiss, Germany). 

### Biochemical analysis (serum urea, creatinine, uric acid and IL-6 levels)

Urea concentration was measured colorimetrically using urea kit (Pars Azmoon, Iran). Creatinine concentration was determined by Jaffe’s method and uric acid concentration was measured by Toos method (18). Where as in MTX groups receiving raspberry fruit extract (specially 200 mg/kg of BW), the AQP1 expression and biochemical analysis (serum urea, creatinine and uric acid) were ameliorated, as compared to the groups which received raspberry fruit extract (100 and 400 mg/kg of BW); Thus serum level of IL-6 was measured only in the group receiving 200 mg/kg of BW Raspberry fruit extract. Serum level of IL-6 was measured by an enzyme-linked immunoabsorbent assay (ELISA) using rat serum IL-6 immunoassay kit. 

### Statistical analysis

Data were presented as the mean ± SEM. 

Statistical comparisons were made using one way ANOVA followed by INSTAT3 and charts was drawn through the EXCEL. Statistical significance inferred at P<0.05. 

### Ethical considerations

All procedures were carried out according to the Guidelines for the Care and Use of Laboratory Animals (National Research Council 1996, Iran). 

## Results

### Histological examination (staining by hematoxylin and eosin)

**Fig.1:**
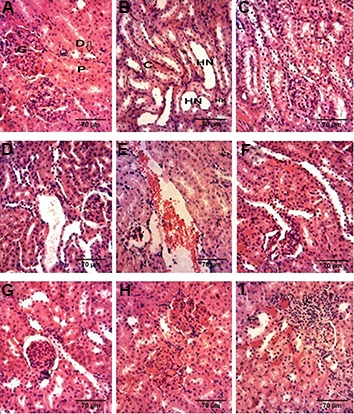
Histopathological studies of kidney. Histological examination of kidney in A, B. Control group shows normal structure, C-F. Methotrexate-treated group shows vacuolization, lymphocytic infiltration, desquamation and hemorrhage. Methotrexate+raspberry extract treated groups tissue demonstrate significantly reduced damages in G. Methotrexate+100 mg rasspberry, H. Methotrexate+200 mg raspberry and I. Methotrexate+400 mg raspberry. P; Proximal convoluted tubule, D; Distal convoluted tubule, G; Glomerulus, C; Collecting tubule, HN; Thin descending limb of Henle, and HK; Thick descending limb of Henleh.

In MTX treated group histological damages such as swelling, vacuolization, lymphocytic infiltration, desquamation and hemorrhage were observed. In the groups receiving mixture of MTX and raspberry extracts the tissue damages were significantly reduced, in comparison to MTX treated group, suggesting the protective effect of raspberry extract (Fig .1). 

### Immunohistochemistry of Aquaporin1 channels 

**Fig.2:**
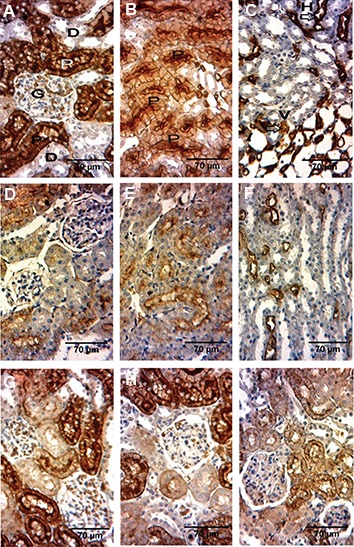
Immunohistochemical localization of AQP1 in the groups. A-C. Control, D-F. Methotrexate-treated, and G-I. Methotrexate+raspberry extract treated groups. AQP1 is expressed in the apical and basolateral A, B. Plasma membranes in the proximal tubule, C. Plasma membranes of the thin descending limb of Henle and in the microvascular endothelium of the outer medullary descending vasa recta. Immunoreactivity for AQP1 was most prominent in the apical membrane of proximal tubules (B), D-F. AQP1 labeling was decreased markedly by the treatment with methotrexate. In methotrexate groups receiving raspberry fruit extracts (G. 100, H. 200, and I. 400 mg/ kg of BW) the AQP1 expression was ameliorated, in comparison with the methotrexate groups. P; Proximal convoluted tubule, D; Distal convoluted tubule, G; Glomerulus, H; Thin descending limb of Henle, and V; Descending vasa recta.

The expression of AQP1 proteins was determined in the kidney by immunohistochemistry. AQP1 was expressed in the apical and basolateral plasma membranes in the proximal tubule (Fig .2A,B), plasma membranes of the thin descending limb of Henle and in the microvascular endothelium of the outer medullary descending vasa recta (Fig .2C). After treatment with MTX (20 mg/kg), the expression of AQP1 was decreased significantly in the modulla and most prominently in the cortex and medulla (Fig .2D-F). 

### Kidney function tests and IL-6 assay 

**Fig.3:**
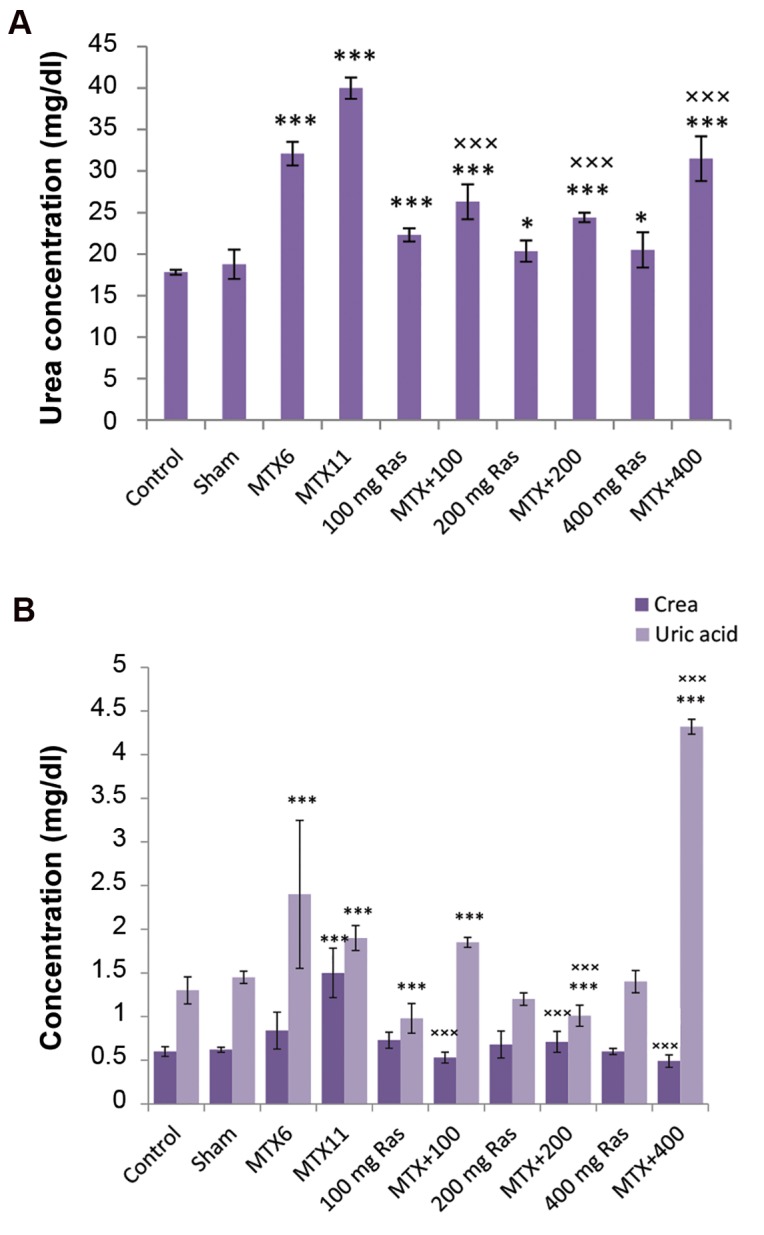
Clinical biochemistry tests (kidney functional tests). Analysis of A. Urea and B. Createnine and uric acid concentrations in blood. Data are presented as mean ± SEM. *; P<0.05, ***; P<0.001, comparison with control group, and ×××; Comparison with methotrexate group.

As shown in Figure 3, MTX treatment increased the serum level of urea, uric acid and creatinine, as compared to the control group (P<0.001), which is an indication of nephrotoxicity. Comparing function of kidneys revealed significant increase in the serum level of urea, creatinine and IL-6, 10 days after injection of MTX in comparison with day six. Using 200 mg/kg of BW raspberry fruit extract for 10 consecutive days, following MTX treatment, significantly decreased the serum level of urea, uric acid and creatinine, compared to MTX treated rats (Fig .3). 

**Fig.4:**
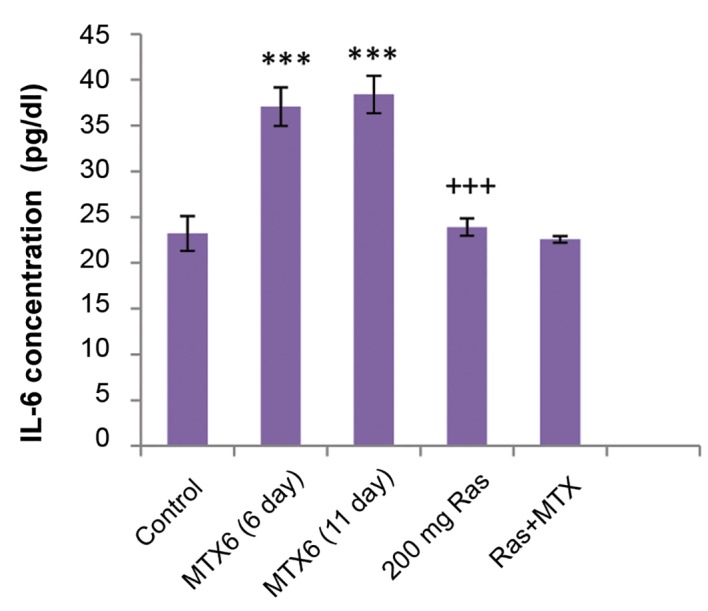
Serum level of IL-6. Methotrexate (MTX) treatment increased serum level of IL-6, in comparison with control group (P<0.001). Using raspberry fruit extract (200 mg/kg of BW, 10 days), following methotrexate, significantly decreased serum level of IL-6, compared to the methotrexate treated rats. ***; P<0.001, comparison with control group, and +++; Comparison with methotrexate group (11 days).

As evidenced in the present investigation, MTX administration resulted in increased serum level of IL-6, indicating the role of this cytokine in drug- induced toxicity, while using raspberry reduced the IL-6 response (Fig .4). 

## Discussion

Kidney is especially vulnerable to toxic insults by several drugs and xenobiotics, because it receives nearly one quarter of the cardiac output. It also transports, metabolizes, and concentrates various toxic substances within its parenchyma (19). Since more than 90% of MTX is excreted via kidneys, nephrotoxicity is one of the most important reasons restricting the use of this drug (3,4). MTX is widely used as a cytotoxic chemotherapeutic agent preventing DNA synthesis, leading to cancer cell death (1,20). However, MTX cytotoxic effect is also harmful to other normal cells and organs in the body. In line with our findings, Kolli et al. (21) previously indicated that MTX administration significantly increased plasma BUN and creatinine levels. 

Renal proximal tubule is a segment of the nephron, which is responsible for the active excretion of toxic chemicals, while it is an important target tissue for many chemicals similar to MTX (4). Thus, it is not surprising to find the most impact of MTX on the proximal tubule function and dysfunction. 

Previous studies demonstrated that MTX administration causes oxidative tissue damage, due to the increased lipid peroxidation and decreased GPX and SOD activities (SOD and GPX are two major anti-oxidation enzymes in the cytosol of living cells) in the kidney. Elevated serum level of the cytokine TNF-alpha and histological analyses demonstrated the severity of the MTX induced systemic inflammatory response. Cytokine IL-6 is released following trauma, endotoxemia and organ injury. Investigations demonstrated that IL-6 signaling has a critical role in the harmful inflammatory process in acute kidney injury and its expression has been observed both in human and experimental animal models (22,23). 

Recent achievements revealed that hydrogen peroxides and oxygen radicals are connected with the development of various pathological processes related to chemotherapy, including adverse effects of anti-tumor drugs. It is currently known that peroxidation injuries will increase antioxidant adaptation in human body. Recent studies demonstrated that MTX administration decreased renal tissue GPX and SOD activity while pentoxyfilline treatment attenuated this suppression (4,18). In addition, profound DNA damages were observed in the renal tubular cells of MTX-treated rats as reflected by the quantitative assessment of TUNEL-positive cells. Kidneys from rats treated with MTX showed marked apoptosis, compared to the control (19). 

The results obtained from immunohistochemistry staining demonstrated that MTX reduced expression of AQP1 in rat kidney. To examine the roles of each mammalian AQPs in the body more directly, Ma et al. (24,25) established knockout mice. Although AQP1 knockout mice were grossly normal in terms of survival and appearance, they were vulnerable to water deprivation and became severely dehydrated. These mice could not concentrate urine suitably. When they were taken away of water for 36 hours, their serum osmolality was increased to a very high level (9). These results suggest that AQP1 is required for the formation of aconcentrated urine (26). It is proposed that lack of AQP1 undermines the countercurrent multiplication process, depending on the rapid equilibration of water across the descending thin limb of Henle’s loops. Subsequent studies have demonstrated that osmotic water permeability of perfused proximal tubules, isolated from AQP1 knockout mice, were only one-fifth of the permeability in proximal tubules dissected from kidneys of normal mice. Chou et al. (27) also showed that the osmotic water permeability of descending thin limb of kidney (cut from AQP1-deficient animals) was decreased by 90%. These studies in AQP1-deficient mice and the subsequent studies in AQP1-deficient humans not only indicated a major importance of AQP1 for water reabsorption in the proximal tubule, but also provided strong evidence that the principle pathway for water reabsorption in the proximal tubule and descending thin limbs is transcellular (via AQP1), but not paracellular (28). The proximal tubule reabsorbs approximately 50-60% of solute and water from the glomerular filtrate. AQP1 is the predominant molecule for water reabsorption in the proximal tubule (24). Kim et al. (29), demonstrated that cisplatin reduced AQP1 expression in kidney. Gao et al. (6), demonstrated that acetazolamid reduced AQP1 expression in kidney. According to their results acetazolamide bonds with AQP1 with six residues as potent binding sites (Ser28, His180, Gly190, Ile191, Asn192, and Arg195), five of which are located in loop E of AQP1. 

Earlier studies have demonstrated the protective effects of anthocyanins against production of cancers *in vitro* and *in vivo* (30). Anthocyanins, presented in blackberries and raspberries, are important for the beneficial health effects associated with their antioxidant, anti-inflammatory, and chemopreventive properties; the biological activity of black raspberry in the prevention of a number of chemically-induced cancers in rodents including esophageal, colon and oral cancer have been demonstrated (16,31). Studies demonstrated that the anthocyanins component in BRB, cyanidin-3-O-glucoside and cyanidin-3-O- rutinoside, selectively inhibited growth, while they stimulated apoptosis of highly tumorigenic cell line obtained from rat esophagus (RE-149 DHD). This effect was increased by adding fresh extract of anthocyanins daily (16). 

## Conclusion

In this study, we used raspberry fruit extract to investigate its effect on MTX treated kidneys. By employing immunohistochemical analysis for AQP1 to assess water reabsorption quality of kidneys and by serological tests to assess kidney function, it is suggested that raspberry fruit extract with its antioxidant components may be a promising drug against MTX-induced cytotoxicity, increasing the AQP1 expression level, and reducing the renal oxidative stress damages. 
